# Some Eye Complications Seen in General Diseases

**Published:** 1904-06

**Authors:** 


### SOME EYE COMPLICATIONS SEEN IN GENERAL DISEASES.*

It is no uncommon thing to find many of the general diseases causing some affection of the eye and frequently the eye trouble is overlooked entirely, especially when it is the interior of the ball that is affected.

Also, we occasionally have our attention first called to some general disease by ocular manifestation of the same:—*e. g.* failing vision due to albuminuric retinitis has frequently been the first symptom leading to an examination of the urine and consequent diagnosis of nephritis, cataract or intractable iritis to a diagnosis of diabetes, choked disk to one of increased intracranial pressure from a tumor, etc. The ocular manifestations are frequently of great service both in the diagnosis and prognosis of many diseases and for these reasons, if for no other, the general practitioner will find it advantageous to have a working knowledge of the ophthalmoscope and at least a comprehensive knowledge of the normal and diseased fundus. Especially will this be found of advantage

*Read by M. H. Bell, M.D., Vicksburg, Miss., before the Warren County Medical Society.

to siphilologists, since in this disease, whether hereditary or acquired, eye complications are frequent.

In hereditary syphilis one of the most frequent manifestations is interstitial keratitis, and when this condition is found in children it furnishes ground for at least a presumptive diagnosis. The same cause also produces, in the young, choroiditis, iritis and retinitis. The acquired form is the cause of many severe ocular manifestations. In rare instances the primary sore is found on the lid or conjunctiva, where it will pursue the same course as elsewhere. In the secondary stage we frequently find an iritis which under proper treatment yields readily and leaves no permanent disability. Later is seen choroiditis, retinitis and optic neuritis followed by atrophy and due to some intracranial lesion. All of these conditions leave the eye more or less disabled, and in none of them does treatment have the same happy result that is seen in iritis. However, by intelligent treatment we can limit the ravages of the disease and in some cases leave the eye practically as good as ever for visual purposes. In choroiditis or retinitis this depends entirely on the location of the inflammation. Another frequent symptom of late occurrence is oculo-motor paralysis due to pressure of a gumma or an exudate on the nerves supplying the muscles of the eye. The prognosis of syphilitic paralysis is usually favorable when we have complete control of the case and can push antisyphilitic medication. Interstitial keratitis has been observed as due to the acquired form, but is rare. Mention might be made of the fact that in the eye diseases due to hereditary syphilis, antisyphilitic treatment rarely ever gives us the brilliant results seen from the same treatment of the acquired form. This, however, should not deter us from carrying out vigorous treatment in the hereditary type of the disease.

The most frequent eye lesion seen as a result of gonorrhea is conjunctivitis, which, as you all know, is a very serious condition. When untreated or improperly treated it usually leads to corneal ulceration, with perforation, infection of the contents of the eyeball and loss of the eye. The disease is more severe in the adult than in the infant. If seen early enough the prognosis is favorable. Other eye conditions due to gonorrhea are iritis, coming on after development of gonorrheal rheumatism, and purulent irido-choroiditis. These affections develop after the disease has been disseminated over the body, and go to prove that gonorrhea becomes a general disease.

Before the days of vaccination, smallpox was one of the most prolific sources of blindness. The eye lesions usually seen were ulcerations of the cornea and conjunctiva.

Diphtheria occasionally attacks the conjunctiva, causing a very severe form of inflammation which is frequently complicated by corneal ulceration and loss of the eye. Coming on at a later date, we see various paralyses of the intra- or extra-ocular muscles, paralysis of accommodation being the most frequent.

Measles frequently has for its first symptom a catarrhal conjunctivitis and, as a later stage in the disease, a phlyctenular conjunctivitis and keratitis.

As a result of scarlet fever we occasionally see a very destructive form of ulceration of the cornea and, rarely, cases of inflammation of the cellular tissues of the orbit. The most important eye lesion found in this disease, however, is albuminuric retinitis brought on by the kidney lesions which result from a good number of cases of scarlatina. Nettleship mentions cases of blindness occasionally seen in a scarlatina in which no demonstrable changes can be found in the eye and which recover entirely in a few days. These are supposed to be due to uremia.

Since the advent of influenza, various eye lesions have been reported as due to it, but considering the very great prevalence of the disease and the rarity of ocular complications, we need lay no great stress upon them. Among them may be mentioned optic neuritis followed by atrophy, orbital cellulitis and paresis of accommodation. This last is seen very frequently after an exhausting disease, and as a result of it we see presbyopes having to get stronger glasses for reading and younger people having to wear glasses for errors of refraction which had never caused any symptoms before the illness. This is an every-day experience with the oculist; patients dating all symptoms of eyestrain or discomfort to some previous illness. I might add in passing that, in my experience, the eye symptoms usually remain; *i. e.*, they rarely ever pass away, even when the patient has otherwise recovered entirely.

Eye lesions due to malarial infection are optic neuritis, followed by atrophy and marginal ulcers of the cornea. Hemorrhage from the retina, due to malaria, has been observed. The optic neuritis, due to malarial infection, must not be confounded with the retrobulbar neuritis due to toxic doses of quinine.

Cerebro-spinal meningitis is frequently accompanied by severe diseases of the visual apparatus: as optic neuritis, thrombosis of the retinal veins and acute purulent choroiditis.

Pyemia and septicemia are sometimes complicated by retinal hemorrhage, thrombosis of the retinal veins and formation of metastatic abscess in the eye.

Typhoid fever is occasionally followed by retrobulbar neuritis, retinal hemorrhage or paralysis of accommodation. In very severe cases we see dessication of the lower half of the cornea with subsequent infection, sloughing and loss of the eye.

A large number of cases of nephritis show eye lesions, various writers giving varying statistics. Probably fifty per cent. of the cases show some eye lesion at some time during the course of the disease and about ten per cent. have albuminuric retinitis. The changes observed in this eye lesion are characteristic and, as previously mentioned, are, in a number of cases, the first symptom leading to a diagnosis of the disease. This form of rhinitis is most frequently observed in chronic interstitial nephritis and in the nephritis of pregnancy. When due to the latter, the physician will have to determine on the propriety of terminating the pregnancy, especially when it occurs in the early months of gestation. When occurring in the chronic interstitial form, it is a grave prognostic symptom: seventy-five per cent. of the patients dying within the first year and ninety per cent. in the second year after the advent of the retinitis.

Diabetes is another disease causing eye lesions in quite a number of cases: among the most frequent are cataract, retinitis, paresis of accommodation, iritis, changes in refraction and optic neuritis. The last leads to diabetic amblyopia, which is a symptom of grave significance. We also see cases of chronic plastic iridocyclitis and keratitis, the latter leading to loss of the eye because of defective nutrition.

Second to syphilis as a cause of iritis we have rheumatism, and we occasionally observe cases of so-called "quiet iritis" due to this disease. This is a form of iritis in which we may have no symptoms calling for an examination of the eye until the whole circumference of the pupillary border of the iris is bound firmly to the anterior capsule of the lens. Other eye lesions found in rheumatism are chronic catarrhal conjunctivitis, with deposits of minute spots of calcareous matter in the conjunctiva, scleritis, episcleritis, keratitis and ocular palsies.

Depending on the scrofulous diathesis we have superficial diseases of the eye which are chronic and are not usually dangerous. Among others I might mention marginal blepharitis and phlyctenular conjunctivitis and keratitis. The phlyctenular inflammations are marked by extreme irritation and sometimes do considerable harm to vision through corneal scars.

It is a fact that these diseases of the eye which are caused by some general disease are frequently overlooked entirely, or are so overshadowed by the symptoms of the causative disease that they are neglected until irreparable damage is done. My object in presenting this paper to you is to refresh your memory on this subject and to remind you of some of the more frequent eye complications which you may expect to see in general practice.—*Mississippi Medical Record.*

